# Transmission of HIV, HBV, HCV in health settings: an anthropological approach on hygiene in Cambodia

**Published:** 2011-01-10

**Authors:** Pascale Hancart-Petitet, Céline Dumas, Anne-Laure Faurand-Tournaire, Sirenda Vong, Alice Desclaux

**Affiliations:** 1 Institut Pasteur du Cambodge, Phnom Penh, Cambodia; 2 Groupe de Recherche Cultures, Santé, Sociétés, Université Paul Cézanne, Aix Marseille, France; 3 Institut de Recherche et Developpement (UMR 145), Dakar, Senegal

## Background

The modalities of HIV, HBV, HCV healthcare-associated infections and the underlying social and cultural logics contributing to this transmission are not precisely known, since hospital hygiene has mainly been studied from a biological point of view until now. However, hospital hygiene is shaped by norms and social-cultural representations, which increase or limit the transmission of infectious agents, always taking place within social relations. In 2006-2009, an anthropological research project (ANRS 12102) aimed at documenting those issues in various health settings in Cambodia. Practices related to hygiene were analyzed from a cultural point of view, especially since norms are interpreted at local level according to social and symbolic logics.

## Methods

We collected qualitative data in formal and informal sectors of care, mainly in general hospital services, maternity wards, primary health centers and in traditional practitioners’ private clinics. We interviewed many participants regarding hygiene practices and social relationships amongst the staff and between health care workers and patients. We also investigated the local representations of hygiene, their impact on the relationships between health care workers and patients and perceptions of transmission risks by health care workers.

## Results

In a context were hygiene practices were limited by the lack of adequate materials and equipments, other factors were identified, which influence and distort hygiene practices. They include: (1) informal and formal social relationships in hospitals, (2) major infection control roles played by cleaners in absence of professional acknowledgment, (3) lack of consideration for hygiene by health professionals that rely on low-ranking staff for hygiene practices. Besides these issues, various questions emerged regarding social science theory. Indeed, doing research on infectious disease transmission led us to include investigations and interpretations related to anthropology of development, historical and social perspectives on public health institutions, and social organization in hospital settings. The social condition of working class (the workers), the legal and illegal systems of care, various aspects related to the politics of reproduction were issues at stake, which leads to more general issues on social changes in Cambodia. Moreover, hygiene issue may be seen as an encounter of the biological body and the social body, whose construction and effects are deeply inscribed in the historical and contemporary forms of social organization and power distribution in Cambodia.

## Conclusion

Our anthropological findings illustrate the importance of comprehensive understanding of hygiene practices; they need to be considered when designing intervention to improve infection control practices in a Cambodian medical setting.

